# Using electronic AKI alerts to define the epidemiology of acute kidney injury in renal transplants

**DOI:** 10.1007/s40620-020-00869-z

**Published:** 2020-12-01

**Authors:** Aled Jones, Jennifer Holmes, Michael Stephens, John Geen, John Williams, Kieron Donovan, Aled O. Phillips

**Affiliations:** 1 Cardiff Transplant Unit, University Hospital of Wales, Cardiff, UK; 2 Welsh Renal Clinical Network, Cwm Taf Morgannwg University Health Board, Pontypridd, UK; 3 Department of Clinical Biochemistry, Cwm Taf Morgannwg University Health Board and Faculty of Life Sciences and Education, University of South Wales, Pontypridd, UK; 4 Institute of Nephrology, Cardiff University School of Medicine, University Hospital, Heath Park, Cardiff, CF14 4XN UK

**Keywords:** Renal transplant, Acute kidney injury, Outcome, Graft survival

## Abstract

**Background:**

Little is known regarding the impact of acute kidney injury (AKI) on renal transplant outcome. Our aim was to define the incidence and outcome of AKI in renal transplant patients using data collected from a national AKI electronic alert system

**Methods:**

The study represents a prospective national cohort study collecting data on 1224 renal transplants recipients with a functioning renal transplant, between April 2015 and March 2019.

**Results:**

Four hundred forty patients experienced at least one episode of AKI giving an incidence rate of 35.4%. Sixty-four point seven% of episodes were AKI stage 1, 7.3% AKI stage 2 and 28% AKI stage 3. Only 6.2% of episodes occurred in the context of rejection. Forty-three point five% of AKI episodes were associated with sepsis. AKI was associated with pre-existing renal dysfunction, and a primary renal diagnosis of diabetic nephropathy. AKI was more prevalent in recipients from a donor after cardiac death (26.4% vs. 21.4%, p < 0.05) compared to the non-AKI cohort. Following AKI, 30-day mortality was 19.8% and overall mortality was 34.8%, compared to 8.4% in the non AKI cohort (RR 4.06, 95% CI 3.1–5.3, p < 0.001). Graft survival (GS), and death censored graft survival (DCGS) censored at 4 years, in the AKI cohort were significantly lower than in the non AKI group (p < 0.0001 for GS and DCGS).

**Conclusion:**

The study provides a detailed characterisation of AKI in renal transplant recipients highlighting its significant negative impact on patient and graft survival.

## Introduction

Acute Kidney Injury (AKI), is associated with increased patient morbidity and mortality [1, 2]. The majority of publications characterizing AKI are dependent on making and recording the diagnosis of AKI through either hospital coding or a retrospective review of hospital records [3–6]. An automated real time electronic (e)-alert system for AKI based on the Kidney Disease: Improving Global Outcomes (KDIGO) change in creatinine diagnostic criteria has been implemented across all areas of the National Health Service in England and Wales, with the aim of facilitating early identification and intervention, and the presumption that this will influence clinical outcomes. To generate the alert the all Wales Laboratory Information Management System (LIMS) (Intersystems TrakCare Lab) automatically compares measured serum creatinine (SCr) values on an individual patient against previous results on the system database. We developed a centralized data collection system based on these alerts, and previously published a comprehensive characterization of the incidence of AKI identified by an electronic alert, and its outcome in the general population of Wales [7–9].

Data related to the incidence and outcome of AKI in the context of renal transplantation are scarce, and in the main rely on making and recording an accurate diagnosis of AKI through hospital coding or retrospective review of hospital records [10–12], and relate to relatively short follow up of patients following transplantation and relatively small patient numbers [13–15]. The most recent data however, using hospital discharge coding data, suggest that the incidence of hospitalisations for AKI among kidney transplant recipients is rising [10]. We have previously demonstrated that in the general population a focus on hospitalised patients with a diagnosis based on retrospective coding data leads to significant under-reporting of AKI compared to electronic AKI alerts [7, 8, 16].

The current study uses a national population-based data set to describe the incidence and outcome of AKI in renal transplant recipients with AKI identified by an automated biochemistry-based electronic AKI alert.

## Methods

Data from all Health boards in the National Health Service in Wales, representing a population of 3.06 million people, was collected from the LIMS on all patients aged 18 yrs or over between 1st April, 2015 and 31st March, 2019 that generated an AKI e-alert. The NHS Number, a unique reference number allocated to patients registered with the NHS in England, Wales and the Isle of Man, was used as the patient identifier to cross reference with the Welsh National Renal database, to identify prevalent transplant patients with and without AKI over the study period. This included any patient with a functioning kidney graft at any time during the study period. Only renal transplant recipients aged 18 years or older with a time since transplantation greater than 90 days were included. The study was approved under the terms of Service Evaluation Project Registration.

The AKI alert is generated by comparing in real time a current SCr value with historic SCr measurements for the same patient. It defines AKI according to KDIGO increase in creatinine parameters [7]. Patients were only included in the study if the AKI alert was generated from a baseline creatinine related to a functioning transplant, i.e. no patients had baseline generated from a creatinine related to a period on dialysis. We have previously demonstrated that this approach ensures collection of all AKI episodes highlighted by an electronic alert across the country, regardless of the clinical location, and excludes patients with end stage renal failure (ESRF), receiving renal replacement therapy (RRT). The AKI Alerts are displayed alongside the biochemical results on the pathology reporting system and consist of one of the following text statements which provide context to the change in creatinine for the receiver:

- Trigger ≥ 26 μmol/l increase in creatinine within 48 h, Associated alert; Acute Kidney Injury alert: rising creatinine within last 48 h.
- Trigger ≥ 50% increase in creatinine within 7 days; Associated alert; Acute Kidney Injury alert: rising creatinine within last 7 day.
- Trigger = 50% increase in serum creatinine against median result for 8–365 days, Associated alert; Acute Kidney Injury alert—creatinine increase over baseline value.

An AKI episode was defined as 30 days, with the first AKI alert defined as the incident alert. Any alert for the same patient within 30 days of the incident alert was not considered a new episode. For patients with multiple episodes, their first episode was defined as their index episode.

Data on patient mortality was collected from the Welsh Demographic Service [17]. Recovery of renal function was defined as a SCr value during the episode no longer in keeping with the definition of AKI when compared to the baseline SCr value associated with the same episode**.** Only surviving patients who had at least one SCr test during the episode were included in the recovery analysis. Pre-study baseline renal function for those transplant recipients with a transplant date of more than 90 days before the study start date of 1st April, 2015 was defined using the last SCr value recorded before the study start date. For all other transplant recipients, the last SCr value recorded before date of transplant + 90 days was used. Post-study renal function was defined as the latest SCr value recorded after the end of the study period. Estimated glomerular filtration rate (eGFR) was calculated using the CKDEpi eGFR formula. A greater than 15% deterioration in the eGFR or a deterioration in eGFR greater than 5 ml/min from the baseline renal function of the patient at the date of entry into the study was used to indicate a significant deterioration in renal function over the course of the study. Only those patients still living with a functioning graft at the end of the study period were included in the analysis of this variable.

Statistical analysis was carried out using SPSS software, version 25 (IBM SPSS, Chicago, IL). Student’s *t* test was used for analysis of normally distributed data. Categorical data were compared using a Pearson Chi-squared test. Kaplan Meier analysis was used to estimate and compare survival of patient groups. Multivariate Binary Logistic Regression was used to assess the association of baseline SCr, AKI stage, Age at AKI, Age at transplant, Recurrent AKI, and Donor type with overall patient survival, overall patient and graft survival, and overall renal recovery from AKI.

## Results

In a total of 1224 renal transplants, 440 patients experienced at least one episode of AKI giving an incidence of AKI of 35.4% over the study period (Table 1). In total there were 937 episodes of AKI with roughly half (224) of the patients who experienced an AKI episode experiencing more than one AKI episode. For patients with multiple episodes, mean number of episodes was 3.2 ± 2.1. The majority (64.7%) of episodes were classified as AKI stage 1 at presentation, with 7.3% AKI stage 2 and 28% AKI stage 3.

**Table 1:** Characteristics of transplant patients who had AKI vs. patients who had no AKI

	AKI	No AKI	p value
Number of patients, n (number of transplants)	440 (440)	771 (784)	
Number of AKI episodes	937	–	
Mean age at time of transplant ± SD (years)	47.2 ± 15.5	46.2 ± 14.7	p = NS
% Female	38.2	35.3	p = NS
Primary renal diagnosis % (n)			
Polycystic kidney disease	12.5 (55)	14.5 (112)	p = NS
IgA nephropathy	11.8 (52)	13.5 (104)	p = NS
Diabetic nephropathy	14.1 (62)	9.6 (76)	p = 0.026
Reflux nephropathy	6.1 (27)	7.1 (55)	p = NS
Glomerulonephritis	10.2 (45)	11.4 (88)	p = NS
Primary FSGS	3.2 (14)	3.4 (26)	p = NS
Hypertensive nephrosclerosis	2.5 (11)	2.7 (21)	p = NS
Idiopathic membranous glomerulonephritis	1.6 (7)	1.4 (11)	p = NS
Other	11.8 (52)	14.1 (109)	
Unknown	9.6 (42)	7.4 (57)	
Diagnosis not recorded	16.6 (73)	14.5 (112)	
Time from transplant to start of study ± SD (days)	1942 ± 2350	1998 ± 2380	p = NS
Mean baseline Creatinine ± SD (mmol/l)	173.0 ± 127.2	128.1 ± 51.2	p < 0.001
Donor type % (n =)			
Donor after brain death	49.5 (218)	44.9 (352)	p = NS
Donor after cardiac death	26.4 (116)	21.4 (168)	p < 0.05
Live related donor	23.6 (104)	33.7 (264)	p < 0.001

The mean age of the AKI cohort of transplant recipients was no different to those with no AKI (47.2 ± 15.4 vs. 46.2 ± 14.7 yrs). There was no difference in gender distribution between the AKI and non-AKI patients (38.2% were female in the AKI vs. 35.3% in the non-AKI group, p = 0.3). Similarly, the average time since transplant to inclusion in the study was also no different between the AKI and non-AKI cohort (1942 ± 2350 vs. 1998 ± 2380 days). In contrast, the mean baseline serum creatinine was significantly higher in the AKI cohort compared to the non-AKI cohort (173.0 ± 127.2 vs. 128.1 ± 51.2 μmol/l). The aetiologies of underlying end-stage renal disease of transplant patients are shown in Table 1. Diabetic nephropathy as a primary renal diagnosis was more common in the AKI cohort. There were no differences in the distribution of all other primary diagnoses.

Within the AKI cohort there were significantly more patients receiving a transplant from a donor after cardiac death (26.4% vs. 21.4%, p < 0.05) and less from live related donation (23.6% vs. 33.7%, p < 0.001) compared to the non-AKI cohort.

**Fig. 1:**
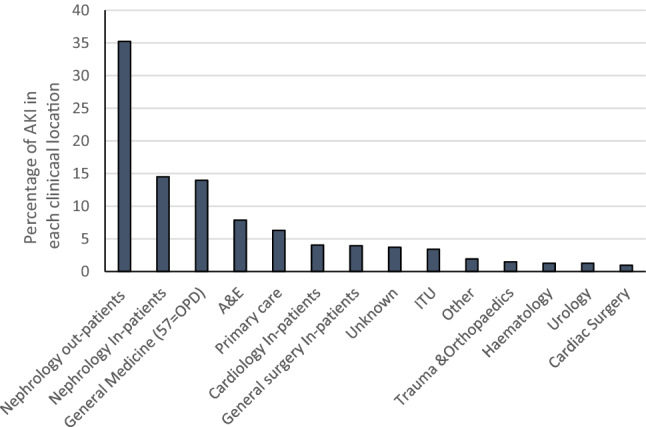
Clinical locations at which the blood test resulting in the AKI alert was generated

The clinical locations of the blood test resulting in the incident AKI alert are shown in Fig. 1. Roughly half of the AKI episodes were associated with an alert related to a nephrology request, the majority of which were a result of a blood test requested in a transplant out-patient setting***.*** Of the remaining AKI episodes, the majority of alerts were reported following requests from general medical in-patients (7.9%), Accident and Emergency (7.9%), primary care (6.3%) and general medical out patients (6.1%).

### Natural history of AKI in renal transplant recipients (Table 2)

Following an AKI episode, 30-day mortality was 19.8% and overall mortality over the study period was 34.8%. Baseline serum creatinine was significantly higher in the cohort of AKI patients who died compared to the surviving patients (210.29 ± 173.1 vs. 153.5 ± 89.2 μmol/l, p = 0.0002). More than one episode of AKI was associated with higher overall patient mortality compared with patients with only one episode (41.1% vs. 28.2%, RR 1.5, 95% CI 1.1–1.9, p = 0.005). In those patients who survived an episode of AKI and had follow-up biochemistry data available, recovery of renal function occurred in 75% of episodes. In the surviving group severity of AKI was a determinant of recovery of renal function, which was lower in patients with incident stage 2 or 3 AKI alerts compared with stage 1 (56.8% vs. 70.5%, P < 0.001). There was no association between non-recovery of renal function and overall patient mortality, with a 29% mortality rate for those that recovered compared to 31.6% for those that did not. There was also no association between non-recovery and repeated AKI episodes, with 52.3% of patients that recovered their index episode experiencing at least one further episode, compared to 60.7% of those that did not recover their renal function in their index episode.

**Table 2:** Natural history of AKI in Renal Transplantation

	Whole AKI cohort	CA-AKI	HA-AKI	p value CA vs. HA-AKI
Number of AKI episodes, n (% of whole cohort)	937	538 (57.4)	273 (29.1)	
Mean age at time of AKI ± SD (years)	55.2 ± 14.7	53.2 ± 14.6	59.6 ± 13.2	p < 0.001
AKI stage 1, % (n)	64.7 (606)	64.5 (312)	78.4 (214)	p < 0.001
AKI stage 2, % (n)	7.3 (68)	7.8 (42)	6.6 (18)	p = n/s
AKI stage 3, % (n)	28.0 (263)	34.2 (184)	18.4 (41)	p < 0.001
Outcome measures				
30-day mortality, % (n)	19.8 (87)	4.8 (26)	16.8 (46)	p < 0.001
Overall mortality, % (n)	34.8 (153)			
30-day recovery of renal function, % (n)	75 (615)	70.2 (340)^	81.8 (185)	p < 0.001
Process measures				
Repeat test within 30 days, % (n)	96.1 (900)	93.9 (505)	98.8 (270)	p = 0.001
Mean time to repeat ± SD (days)	3.8 ± 5.9	5.5 ± 7.13	1.43 ± 1.87	p < 0.001

Recovery of renal function included only surviving patients with available tests of follow-up renal function: 710 episodes were included in the 30-day recovery of renal function analysis (484 episodes, CA; 226 episodes, HA-AKI)

Multivariate Binary Logistic Regression showed that a lower baseline SCr (B = − 0.01, p < 0.001) and age at AKI (B = − 0.07, p < 0.001) were associated with an increased likelihood of overall patient survival, and patients with multiple AKI episodes were less likely to survive compared to those who had a single episode (OR = 0.47, 95% CI 0.30–0.75, p < 0.001). Similarly, a lower baseline SCr (B = − 0.01, p < 0.001) and age at AKI (B = − 0.07, p < 0.015) were also associated with an increased likelihood of overall patient and graft survival. Patients with AKI stage 3 were less likely to survive with a functioning graft compared to those with AKI stage 1 (OR = 0.46, 95% CI 0.25–0.86, p = 0.014), as was the case with patients with multiple AKI episodes compared to patients with a single episode (OR = 0.39, 95% CI 0.25–0.61, p < 0.001). Furthermore, a lower baseline SCr (B = − 0.01, p = 0.048) lower age at AKI (B = − 0.05, p = 0.011), and higher age at transplant (B = 0.05, p = 0.011) were associated with an increased likelihood of overall renal recovery from AKI, and patients with multiple AKI episodes were far less likely to recover compared to those who had a single episode (OR = 0.28, 95% CI 0.17–0.47, p < 0.001).

Our previous work in non-transplant associated AKI has demonstrated that a significant proportion of patients highlighted with an AKI alert do not have further monitoring of renal function. In this transplant recipient cohort 96.1% of the AKI episodes were associated with a repeat measure of renal function within 7 days of the alert, with a mean time to repeat of 3.8 ± 5.9 days. It should be noted however that in 37 episodes no repeat measure of renal function was requested within 7 days of the incident alert.

The clinical diagnosis associated with each AKI episode is shown in Table 3. Rejection was associated with only 6.2% of all episodes. This cohort was significantly younger than the non-rejection group, and had a higher proportion of AKI stage 3 at presentation. In the non-rejection cohort, the predominantly associated clinical diagnosis was sepsis, with urinary tract and respiratory infection accounting for the majority of cases. There were no differences in mortality between the rejection- and non-rejection-associated AKI episodes. Recovery of renal function was however significantly worse following rejection-associated AKI, reflecting the higher proportion of stage 3 AKI at presentation.

**Table 3:** Clinical course by clinical diagnosis associated with AKI episode

	Number of episodes (%)	Mean age ± SD (years)	AKI stage (% of episodes)	30-day mortality, % (n)	30-day recovery of renal function, % (n)
Non-rejection, n (%)	692 (73.9)	56.11 ± 14.5	1: 67.5 2: 9.1 3: 23.4	9.39 (65)	79.7 (486)
Sepsis, n (%) (Urinary) (Respiratory)	408 (43.5) (171) (11)	56.38 ± 14.63	1: 67.9 2: 10.3 3: 21.8	8.3 (34)	89.2 (364)
Dehydration	142				
Cardiac	27				
Obstruction	16				
Recurrent disease	14				
Contrast	7				
Other	65				
Rejection, n (%)	58 (6.2)	44.91 ± 16.3*	1: 53.4* 2: 3.4 3: 43.1*	5.17 (3)	49.06 (26)

Details of clinical diagnosis associated with AKI were not available for 187 episodes. Mortality data were available for 692 non-rejection episodes and 58 rejection episodes. Recovery of renal function included only surviving patients with available tests of follow-up renal function: for non-rejection AKI recovery included 608 episodes and for rejection 53 episodes. For the sepsis-associated AKI group mortality data were available for all 408 episodes, recovery of renal function included 364 episodes

*p < 0.001 compared to Non rejection AKI

### Comparison of hospital- and community-acquired (HA)/(CA) transplant-associated AKI

CA-AKI accounted for 57.4% of all episodes (n = 538), of which hospitalisation following the alert occurred in only 37 episodes. Transplant out-patients’ requests accounted for 61.3% of CA-AKI. The other major sources of CA-AKI alerts were Accident and Emergency (13.9%), Primary care (11.0%) and medical out-patients (10.6%).

HA-AKI accounted for 29.1% (273) of all transplant-associated AKI. For hospital-acquired AKI, the largest single cohort was reported following a blood test requested from the renal transplant in-patient ward (49.8%), followed by general medical in-patients (27.1%), cardiology in-patients (13.9%), general surgery in-patients (13.5%), and intensive treatment unit (ITU) (11.7%). The remaining 13.4% (126) of alerts were generated in an in-patient setting, but as no results were available for the previous 7 days it was not possible to confidently classify these as either CA- or HA-AKI.

The proportion of incident AKI alerts reported as AKI stage 3 was significantly higher in CA-AKI compared to HA-AKI (Table 2). Conversely the proportion of AKI stage 1 was lower in the CA-AKI group compared to HA-AKI. Compared to CA-AKI, 30-day mortality was significantly higher for patients following HA-AKI (HA-AKI: 16.8% vs. CA-AKI: 4.8%, p = 0.001). In contrast to mortality outcomes, for the surviving patients recovery of renal function at 30-days was significantly better following HA-AKI (HA-AKI; 81.8% vs. CA-AKI: 70.2%, p < 0.001). Within the CA-AKI cohort the mean time to repeat measurement was 5.5 ± 7.1 days; following 33 AKI episodes there were no repeat measures of renal function within 7 days of the alert. In contrast, in the HA AKI cohort, there were no repeat measures of renal function within 7 days following only 3 AKI episodes, and the average time to repeat was significantly shorter than in CA-AKI (1.4 ± 1.9 days, p < 0.001).

### Influence of AKI on transplant patient outcomes (Table 4)

**Fig. 2:**
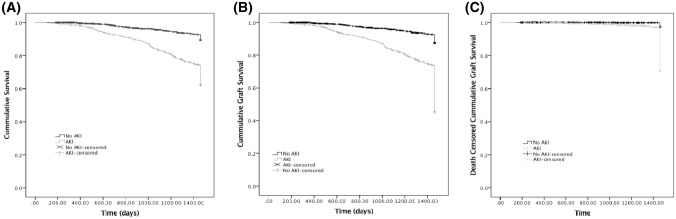
Impact of AKI on patient and graft survival. **a** 4 year censored survival of renal transplant patients experiencing at least one AKI episode compared to renal transplant recipients with no episodes of AKI during the study period. Renal graft survival (**b**) and death censored renal graft survival, both censored at 4 years, in patients experiencing at least one AKI episode compared to renal transplant recipients with no episodes of AKI during the study period

Mortality censored at 4 years was significantly higher in the AKI cohort compared to those who did not have an AKI episode during the study period (p < 0.0001, Fig. 2a). Overall mortality for the non AKI cohort was 8.4% compared to 34.8% in the AKI group (RR 4.1, 95% CI 3.1–5.3, p < 0.001 compared to AKI cohort).

**Table 4:** Comparison of outcomes AKI vs No AKI in renal transplantation

	AKI	No AKI	p value
Number of patients	440	771	
Mean duration of follow up since Transplant ± SD (days)	3669.6 ± 2701.7	3515.7 ± 2780.3	p = 0.34
Overall mortality, % (n)	34.8 (153)	8.6 (66)	p < 0.001
Patient status at end of study			
Living with functioning graft	50.2 (221)	91.6 (706)	p < 0.001
Living with non-functioning graft	15.0 (66)	1.6 (12)	p < 0.001
Died with functioning graft	30.0 (132)	8.3 (64)	p < 0.001
Died with non-functioning graft	4.8 (21)	0.3 (2)	p < 0.001
Renal function at end of study			
Creatinine mmol/l (mean ± SD)	167.6 ± 82.4	123.8 ± 50.7	p < 0.001
eGFR (ml/min) (mean ± SD)	42.3 ± 20.7	53.9 ± 17.3	p < 0.001
% with significantly worse renal function	57	24.5	p < 0.001

927 patients were included in analysis of the renal function at the end of the study variable (AKI, 221; No AKI, 706)

A comparison of the status of the patients at the end of the study period demonstrated significantly fewer patients alive with a functioning graft in the AKI group. More patients were alive with a non-functioning graft, and a higher proportion of patients had died, either with a functioning graft or with a non-functioning graft, in the AKI group. The association between AKI and graft failure was analysed by Kaplan–Meier estimation. Graft survival (GS), and death censored graft survival (DCGS) censored at 4 years, in the AKI cohort were significantly lower than in the non AKI group (p < 0.0001 for both GS and DCGS, Fig. 2b and c).

For patients who had poststudy renal function data available (i.e. alive with a functioning graft) the pre-study baseline renal function was no different in the AKI group compared to the non-AKI group (133.4 ± 52.0 μmol/l vs. 123.9 ± 17.3 μmol/l). In contrast, post-study serum creatinine was significantly higher in the AKI cohort compared to the non-AKI cohort (167.6 ± 82.4 μmol/l vs. 123.8 ± 50.7 μmol/l, p < 0.001). Similarly, whilst the eGFR was not significantly different at the beginning of the study (50.5 ± 18.1 ml/min for the AKI cohort vs. 53.9 ± 17.3 ml/min for the non-AKI cohort), at the end of the study period those from the AKI cohort had a significantly lower eGFR compared to the non-AKI group (42.3 ± 20.7 ml/min vs. 55.4 ± 20.1 ml/ml, p < 0.001). The percentage of patients with an end of study period renal function which was worse than the starting renal function as defined by a greater than 15% or 5 ml/min deterioration in eGFR, was also higher in the AKI group (57.0% vs. 24.5%, RR 2.3 95% CI 1.9–2.7, p < 0.001). The duration of follow up from the time of transplant was no different between these two groups (3669.6 ± 2701.7 days for the AKI group vs. 3515.7 ± 2780 days for the non-AKI group, p = 0.34).

## Discussion

Studies on AKI in renal transplant recipients are scarce but report a 40–50 fold higher incidence than the general population [11, 13], and occurring in up to 85% of hospitalised renal transplant patients [14]. It is postulated that the nature of AKI in renal transplants may be different to that seen in the general population with different susceptibilities related to denervated kidneys, susceptibility to haemodynamic instability, use of nephrotoxic drugs, especially calcineurin inhibitors, immune-related injury and predisposition to opportunistic infections. Whilst numerous published studies have described acute renal dysfunction in the immediate post-transplant phase [15, 18–22], very little is known of the nature and impact of AKI in the “maintenance” phase of renal transplantation. To address this, our focus was on AKI beyond the first 90 days of transplantation and well into the maintenance phase of prevalent renal transplant patients.

Our data report an incidence of AKI of 35%. In contrast, Mehrota et al. reported an AKI incidence of 11.3% in transplant patients in a study confined to hospitalised patients only, and identified AKI using coding data [11]. Our higher incidence may in part be explained by the high incidence of non-hospitalised AKI in renal transplant patients that we have identified but not previously reported. In contrast, a 51% incidence rate of transplant-associated AKI was reported by Nagarajan et al. [12]. That study focused on a relatively short term period of follow up, with AKI occurring predominantly in the first year of the follow up period. This higher reported incidence may therefore reflect the greater burden of AKI in the early phase following transplantation compared to our data which are of AKI occurring later in the phase of the prevalent transplant population. Nakamura et al., in contrast, reported an AKI incidence of 20.4% in transplant recipients, which is significantly less than our reported incidence. In this study, with a mean follow up period of four years post-transplantation, the majority of the AKI occurred within two years of the transplant [13], and AKI was only identified through nephrology/transplant clinics. Our non-selective approach, which identified all AKI based on every blood test that a prevalent renal patient had at any location, highlights that such an approach will significantly underestimate the true incidence of transplant-associated AKI.

In terms of clinical outcome, whilst AKI in the context of renal transplant carries a significant short term (30-day) and longer term mortality, this is similar to our previously reported data in the adult population [7, 23, 24], suggesting there is no excess in mortality when AKI occurs in renal transplant recipients compared to the general population. Our data also demonstrate that poor renal function prior to an AKI episode is associated with higher mortality. Although renal function in the immediate period following the AKI episode recovered in three-quarters of cases, AKI impacted negatively on graft survival and function, with a higher rate of graft loss, and significantly worse renal function in surviving patients in the AKI cohort. This is consistent with the previously published hospital-based, single centre and relatively small studies suggesting an association between AKI and risk of transplant loss [11–14]. Although the mechanistic link between AKI and graft loss remains speculative, it has been proposed that an episode of AKI may up-regulate inflammatory and fibrotic signalling pathways, leading to progressive structural kidney damage [25–28].

The only demographic differences between our AKI and non-AKI cohort was a higher prevalence of diabetic nephropathy as the cause of ESRF, and a higher serum creatinine at baseline. This is similar to the findings of the studies by Nakamura et al. [13] and Mehrotra et al. [11] demonstrating an association between post-transplant AKI, renal function and diabetes. For the general population, both diabetes [29–32] and CKD [33, 34] have previously been described in the literature as risk factors for AKI. In this context at least, our data would suggest the transplant population is therefore similar to the general adult population in terms of AKI risk. The higher baseline SCr in the AKI cohort along with the fact that patients with AKI had a conceivably higher prevalence of chronic graft dysfunction and a higher prevalence of the related alloimmune and non-alloimmune causes of chronic graft dysfunction suggest AKI may represent more a marker of clinical frailty rather than playing a direct pathogenetic role towards the risk of death and graft failure. It is of note that the prevalence of a kidney from a deceased donor was higher in our AKI cohort. This is consistent with previous published data highlighting deceased donor transplant to be a significant risk factor for the development of AKI [12]. This may be related to the incidence of delayed graft function and resultant renal impairment in this cohort, although this remains speculative as we were unable to report on delayed graft function in our study.

In the non-transplant population, in the majority of cases, AKI does not reflect intrinsic kidney disease but is rather a complication of other primary illnesses. Our data suggest that this is also reflected in transplant patients. In this study AKI in the context of the maintenance phase of renal transplant does not represent either rejection or recurrence of the primary renal disease. The majority of cases are associated with a clinical diagnosis of sepsis, with roughly a half identified via a non-nephrology/transplant request. This is consistent with the work of Nakamura et al. demonstrating that bacterial infections and predominantly urinary tract infections were the most common aetiological factors contributing to renal-transplant-associated AKI, although this study was confined to a small number of living donor recipients alone and in the setting of the outpatient nephrology/transplant clinic [13]. This pattern of AKI is however different to that reported in the early post operative period when the nephrotoxic effect of immunosuppressive agents, in particular calcineurin inhibitors and rejection, are more common [15].

Our data demonstrate that over half of transplant-associated AKI is detected in the community, with only a small minority of cases being admitted to hospital. These cases therefore would not be reported in studies based on hospitalisation, nor would AKI identified through hospital coding. Although HA-AKI in this study represents roughly a third of the AKI cases, it should be noted that there were also a significant number of episodes classified as ‘undetermined in hospital alerts’, as these patients, whilst alerting in an in-patient setting, had no results for the previous 7 days. Our data therefore likely reflect a significant underestimation of the true incidence of HA-AKI in renal transplant recipients. Mortality following HA-AKI in this transplant cohort was significantly higher than following CA-AKI. This higher mortality is not a reflection of AKI severity per se*,* as there was a higher proportion of incident AKI stage 3 alerts in the CA-AKI group. The higher mortality in the HA-AKI cohort again mirrors our previous data in the general adult and paediatric populations [9, 35]. As the majority of AKI cases do not represent intrinsic kidney disease it is likely that the excess mortality in HA-AKI reflects the severity of the primary illness precipitating AKI. Previously we have demonstrated that in the general adult and paediatric populations, in those surviving an AKI episode, renal function is better following HA- compared to CA-AKI [7–9, 16]. In part at least this reflects clinical inactivity and a failure to recognise the importance of the alert. This was supported by the lower number of patients with CA-AKI compared to HA-AKI who have a repeat measure of creatinine even following severe AKI, and a longer time to repeat for those who do have a repeat measure. It is of note that few patients in the transplant cohort did not have any follow-up bloods, however, as in the general non-transplant adult population, in this study of transplant patients the time to repeat a measure of renal function was significantly longer in the CA-AKI group which may reflect a slower response time that may then in turn result in later initiation of interventions which may facilitate recovery of renal function.

Although this study is to our knowledge the first national study using an e-alert-based system to characterise the magnitude and impact of AKI in renal transplant recipients, its findings need to be qualified by its limitations. As the e-alert system is IT driven it lacks “intelligence” and therefore there is no clinical context applied. Using an IT-based approach also excludes patient clinical information, such as patient co-morbidities, medication. Linkage to comorbidity data in particular would have helped strengthen the suggestion made by this study that AKI may represent more a marker of clinical frailty rather than playing a direct pathogenetic role towards the risk of death and graft failure. We are also unable to generate linkage to primary care data sets. As a consequence, a detailed analysis of the clinical response to the AKI episode cannot be captured. Our data also lack details on the need for RRT, and do not shed light on the cause of death. Our data report the incidence of AKI in which the diagnosis is a creatinine-based definition in which the baseline creatinine is generated by the patients’ historical results. As such, this may not meet the strict agreed AKI definition of “abrupt deterioration”, and does not take into account a “urine output”-based AKI diagnosis. Despite these limitations our study provides a detailed characterisation of AKI in renal transplant recipients and highlights its impact on patient and graft survival.

## Data Availability

The data that support the findings of this study are available on request from the corresponding author.
